# Cross-reactivity and localization of *Schistosoma mansoni* antigen for immunodiagnosis

**DOI:** 10.1590/1414-431X2025e15126

**Published:** 2026-01-09

**Authors:** Fatemah E. Alajmi

**Affiliations:** 1 Department of Biology, College of Science, University of Hafr Al Batin, Hafr Al Batin, Saudi Arabia

**Keywords:** Schistosomiasis, Cross-reactivity, Life cycle stages, Monoclonal antibodies, Immuno-phosphatase, Immuno-peroxidase

## Abstract

Schistosomiasis remains a major global health concern, affecting approximately 260 million people, with 440 million experiencing morbidity and over 800 million at risk of infection. According to the World Health Organization (WHO), it ranks as the second most socioeconomically impactful infectious disease and the third most significant parasitic disease in terms of public health. This study aimed to improve immunodiagnostic tools for *Schistosoma mansoni* in resource-limited settings by developing monoclonal antibodies (MoAbs) using locally available materials. MoAbs were produced using hybridoma technology and assessed for specificity to soluble worm antigen preparation (SWAP) through ELISA. Immuno-phosphatase and immuno-peroxidase staining were employed to localize target antigens across various life cycle stages and assess cross-reactivity with related species. Four distinct MoAbs demonstrated strong phosphatase and peroxidase activity in the gut and tegumental tubercles of *S. mansoni* adult worms, with extreme (4+) phosphatase staining. The dorsal tubercles and oral/ventral suckers showed strong (3+) peroxidase staining. *S. mansoni* schistosomula showed positive staining in the oral sucker and penetration glands, while cercariae showed no reactivity. Cross-reactivity with *S. haematobium* was minimal, showing only weak (1+) peroxidase staining in gut and tegumental structures, as well as the intact worm's tegumental tubercles and suckers. In conclusion, the MoAbs developed exhibited high specificity for *S. mansoni* with limited cross-reactivity to *S. haematobium*, supporting their potential utility in locally produced, sensitive immunodiagnostic tools to strengthen schistosomiasis control and elimination efforts in endemic regions.

## Introduction

Schistosomiasis is a neglected tropical disease identified by the WHO (1) and primarily exists in tropical and subtropical countries. This helminthic disease, caused by dioecious digenean schistosomes within platyhelminthes or flatworms, affects vertebrate hosts in more than 70 countries. Active infections affect 260 million people, while 440 million suffer from residual morbidity, and nearly 800 million individuals are at risk of infection. It occurs in many developing countries in tropical Africa, the Middle East, Asia, and Latin America. *Schistosoma mansoni* belongs to the class Trematoda, which has a fluke-like appearance. This species is the most prevalent schistosome that infects humans and causes gastrointestinal infectious diseases (2,3).

Schistosomiasis exhibits a unique three-phase immune response, which corresponds to key changes in the parasite's life cycle. The parasite undergoes a series of stages, beginning with cercariae that penetrate the skin and develop into a schistosomula. These schistosomula infiltrate the vasculature and travel to the lungs via the pulmonary artery. Finally, they are expelled from the lung capillaries, mature into males and females, pair up, and become impervious to host immune reactions. They migrate to the peri-vesical veins (*S. haematobium*) or mesenteric venules (*S. mansoni and S. japonicum*) upon reentering venous circulation (4). The acute phase occurs between 4 to 8 weeks after infection, when individuals may experience abdominal pain and fever. Hundreds to thousands of ova, which can survive for three to ten years, can be laid by worms. The primary cause of schistosomiasis is the trapping of eggs in the liver or outside the body through urine (*S. haematobium*) or stool (*S. mansoni* and *S. japonicum*). Over time, chronic inflammation, fibrosis, and severe organ injury are concomitant with continuous egg deposition, which induces a granulomatous response (3,5).

Schistosomiasis immunobiology is characterized by intricate, multifactorial processes that involve numerous surface antigens and excreted parasite molecules. Furthermore, it appears that the primary humoral immune response to schistosomes is directed toward glycan epitopes on glycoconjugate antigens at various life cycle phases, and a significant proportion of the molecules that regulate the interaction with the host immune response. Alternative diagnostic methods, such as antibody-based assays, have been developed to overcome the low sensitivity of parasitological tests and the fact that egg excretion occurs approximately 40 days post-infection. Improving serological diagnostic tests depends on identifying specific schistosome antigens with high sensitivity to detect even mild infections (6,7).

Traditionally, B cells are known to contribute to immune responses through the production of antibodies. However, the regulatory properties of B cells have recently been documented in numerous inflammatory diseases. Breg cells have immunosuppressive functions through a variety of regulatory mechanisms. In addition to schistosome-induced splenic B cells, B-1a cells from the peritoneal cavity also possess the regulatory ability to induce Treg cells. Additionally, it has been proposed that B cells are crucial for the Th2 response during *S. mansoni* infection. This suggests that B cells play a multifaceted role in regulating immunity against schistosome infection. In general, antibody assays report mainly immunoglobulin G (IgG) and, infrequently, immunoglobulin M (IgM). These assays are designed to detect impure antigen extracts, such as cercarial secretions, schistosome egg antigen (SEA), and soluble worm antigen preparations (SWAP), or to detect purified or recombinant antigens. Hybridoma cell cultivation in serum-free media is better for generating pure MoAb, leading to reduced non-specific reactions in diagnostic tests (8,9).

A significant factor contributing to the lack of disease control is the utilization of inefficient diagnostic methods to detect infection and evaluate the effectiveness of control measures. In contrast to coprological diagnostic tools, immunodiagnostic tools based on schistosome antigens are less time-consuming and exhibit higher sensitivity. These assays have emerged as promising alternatives for the surveillance of schistosomiasis, particularly in regions where the disease has been virtually eradicated (10). The present study aimed to test the cross-reactivity of four selected MoAb constructed from *S. mansoni* infection and post-injection with the SWAP antigen. Reactivity was tested in response to intact sections of worms, schistosomula, cercariae, and intermediate snails of *S. mansoni*. It also includes intact sections of worms and intermediate snails of *S. haematobium*.

## Material and Methods

### Parasites and experimental animals

The Schistosome Biological Supply Center (SBS) at the Theodor Bilharz Research Institute (TBRI), Egypt, provided parasitic and animal materials. *Biomphalaria alexandrina* and *Bulinus truncatus* snails were used to maintain Egyptian isolates of *S. mansoni* and *S. haematobium* as primary hosts, and Swiss mice (∼20 g each) and hamsters (∼100 g each, used only to maintain the *S. haematobium* life cycle for cross-reaction) as secondary hosts, respectively. Both animal species were females aged 6-8 weeks. The different life cycle stages were identified, embedded in paraffin, sectioned, and stained. Ethics approval and animal considerations were covered under the TBRI Institute's clinical veterinary research roles. They follow the internationally agreed-upon ethical best practices in clinical veterinary research (CVR) as outlined in the Helsinki Declaration (11,12).

### Production of monoclonal antibodies

At six weeks of age, 5 female Balb/c mice (Monoclonal Antibody Production Unit, TBRI, Egypt) were infected with 50 *S. mansoni* cercariae and post-boosted with 200 µg *S. mansoni* SWAP antigen at 4 weeks of infection to increase reactivity against schistosome antigens. The SWAP antigen was prepared by suspending adult *S. mansoni* worms in 0.01 M phosphate-buffered saline (PBS) at pH 7.2, homogenizing, centrifuged (at +4°C at 6,000 *g* for 15 min), and the supernatant was collected. The protein content of the supernatant was determined and stored at -20°C until needed. Sensitized spleen cells were extracted 8 weeks after infection and fused with non-secreting murine myeloma cells (P3X63Ag8-U1) using 43% polyethylene glycol (Sigma Chemicals, USA) according to Greenfield (13). After selection in hypoxanthine-aminopterin-thymidine (HAT) medium, hybridomas were screened for the most reactive antibodies against SWAP using indirect ELISA. Five mice were intraperitoneally injected with highly reactive cell lines. Each mouse was injected with one cell line to induce ascites (13-15). After ascites production, purification, and concentration, the antibodies were rescreened again using ELISA, then designated as Mo1Ab, Mo2Ab, Mo3Ab, and Mo4Ab for easy manipulation and labeling. Each monoclonal antibody (Mo1Ab) was obtained from a single mouse injected with a single cell line.

### Histological investigations

Formalin-fixed and paraffin-embedded blocks were prepared and sectioned for different schistosome life cycle stages. These stages were supplied through SBS of TBRI, where they are routinely available. Hematoxylin-eosin (HE) staining was performed to illustrate the histological structure of *S. mansoni* and *S. haematobium* worms (gut, parenchyma, tegumental tubercles, and muscularis). Intact worms stained with HE show the tegumental surface, gynecophoric canal, and oral and ventral suckers. Intact *S. mansoni* cercariae, schistosomula, and the biforked tail of cercaria after transformation were also stained with HE, as were sections of *Biomphalaria alexandrina* and *Bulinus truncatus* snails. An Optika binocular microscope (Italy) with an adjusted digital camera was used to capture the photomicrographs.

### Immuno-phosphatase/immuno-peroxidase staining

#### Pretreatments of tissue sections

The free sites of tissue sections were occluded with 0.3% nonfat milk and incubated at room temperature for 30 min after hydration in descending grades of ethyl alcohol to inactivate endogenous alkaline phosphatase. Endogenous peroxidase was inactivated by incubating tissue sections with methanol containing 3% H_2_O_2_ for 5 min after hydrating them in descending grades of ethyl alcohol.

#### Staining procedures

The sections were washed with Tris buffer-Tween three times (each for 5 min). Next, various MoAbs were administered at a 1:100 dilution for 1 h, and the slides were washed as previously described. The sections were incubated with alkaline phosphatase-conjugated goat anti-mouse IgG (Sigma, USA) diluted at 1:500 for one hour, and then the BCIP/NBT substrate was added for 30 min (for immuno-phosphatase staining). The slides were incubated with horseradish peroxidase-conjugated goat anti-mouse IgG (Sigma) diluted at 1:500 for 1 h, and the substrate (3'-amino-9-ethylcarbazole, Sigma) was added to 30% H_2_O_2_ for 30 min (for immuno-peroxidase staining). The sections were counterstained with Mayer's hematoxylin and then mounted in glycerol. All procedures were conducted in a humidified chamber at ambient temperature (16,17).

### Data analysis

Schistosomal antigen localization in sections through different life cycle stages was counted and scored based on photomicrography. -ve: no reaction, 1+: weak reaction, 2+: moderate reaction, 3+: strong reaction, and 4+: extreme reaction. At least three sections from each schistosomal stage were examined and scored visually by an expert in schistosomal pathology.

## Results

### Morphological structure of *S. mansoni* worms

**Figure 1:**
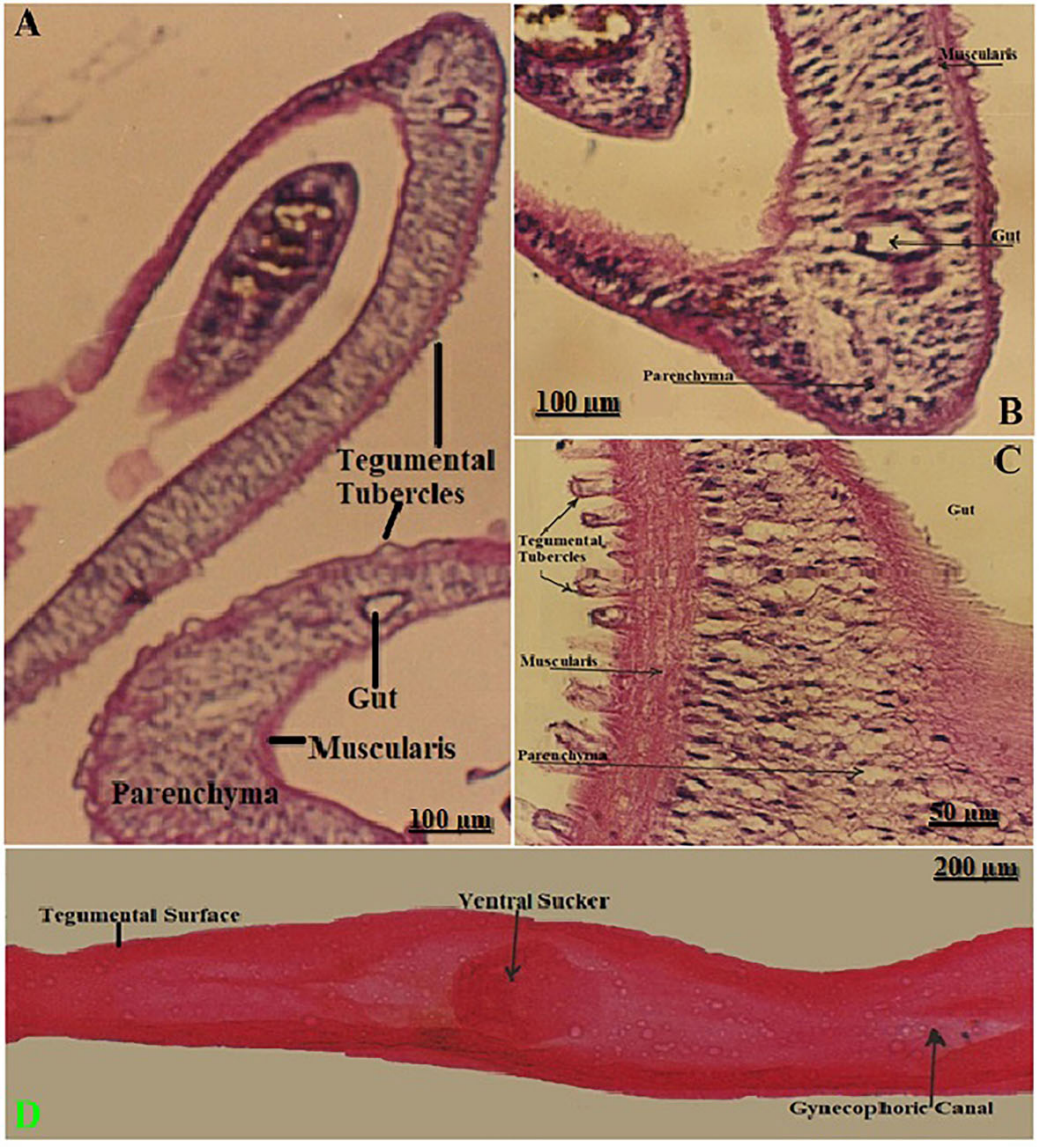
Histological sections of *S. mansoni* worms stained with HE, showing the internal anatomy, including the gut, parenchyma, muscularis, and prominent tegumental tubercles (**A** and **B,** 100×; **C,** 400×). **D**, Whole-mount longitudinal section (40×) of an intact male worm showing the tegumental surface, ventral sucker, and gynecophoric canal. The tegumental surface is crucial for host-parasite interactions, while the gynecophoric canal is used to harbor the female during copulation. Scale bars: 200, 100, and 50 µm.

Histological sections of *S. mansoni* worms stained with HE are shown in Figure 1A-C. The tegumental tubercles were prominent in all sections, indicating active structural and immunological roles at the host-parasite interface. The gut showed a clear lumen and surrounding epithelial cells, confirming functionality in nutrient uptake. The muscularis layer was well-defined, suggesting its role in motility and shape maintenance. The parenchyma appeared loosely packed and supportive, providing the internal matrix for various organs. The intact worm showed a tegumental surface, the gynecophoric canal (hallmark of the male worm's anatomy, essential for reproduction), and ventral suckers (Figure 1D).

### Reactivity of *S. mansoni* MoAb within different tissues of *S. mansoni* worms

A panel of *S. mansoni* MoAb, the Mo1Ab, Mo2Ab, Mo3Ab, and Mo4Ab, was used to localize schistosomal antigens within different parasite tissues. The present study was performed using paraffin-embedded sections of formalin-fixed as well as intact worms. Myeloma cell ascites were used as the negative control. At least three sections from each schistosomal stage were stained with one MoAb to confirm its reactivity.

### Paraffin sections

**Figure 2:**
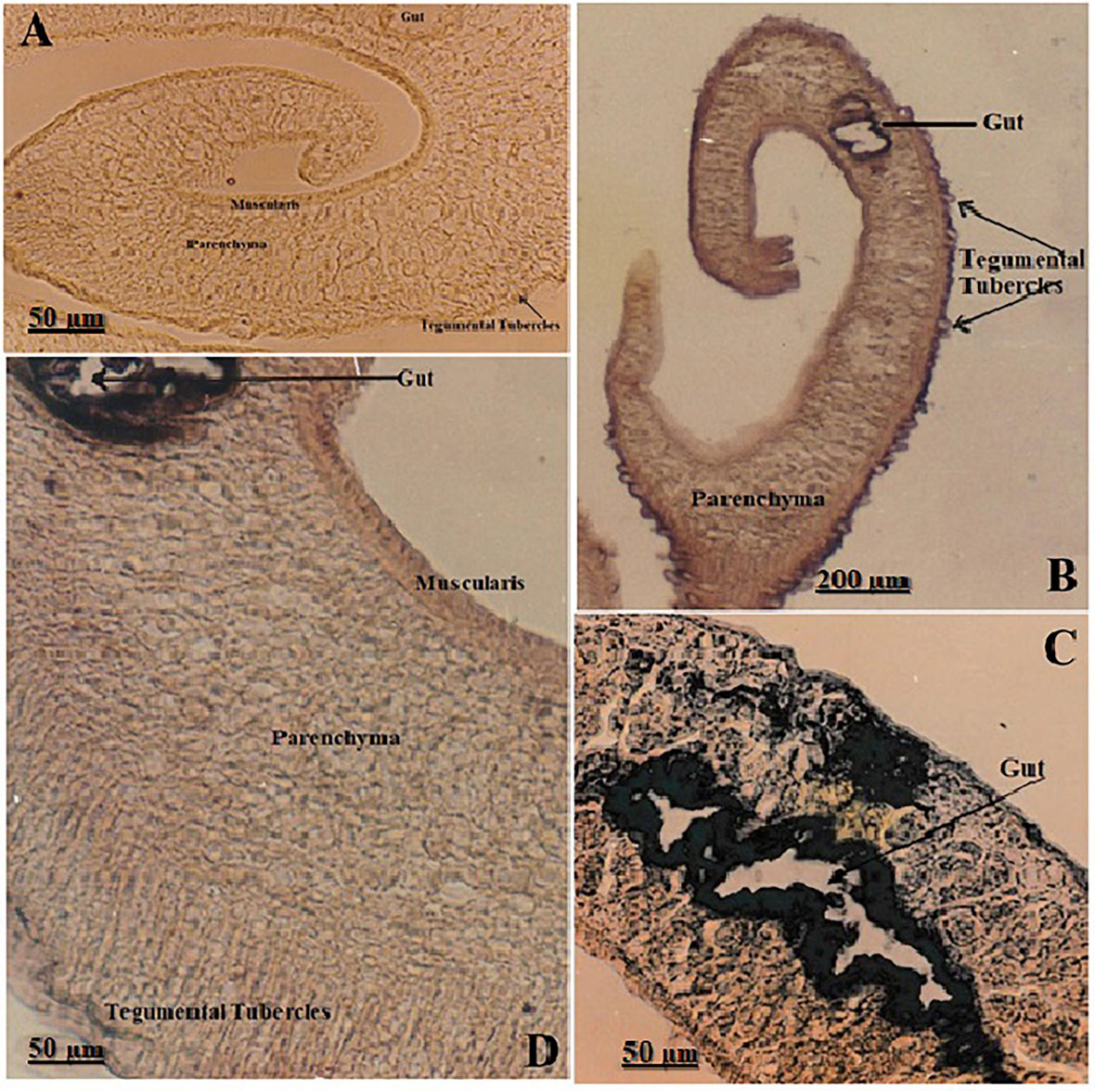
Sections of *S. mansoni* worms subjected to phosphatase immunostaining. **A**, Negative control showing no phosphatase reactivity in the gut, tegumental tubercles, muscularis, or parenchyma (400×). **B**, Positive phosphatase activity in the gut, tegumental tubercles, and parenchyma (monoclonal antibody Mo2Ab, 40×). **C** and **D**, High magnification views (400×) revealing variable phosphatase staining patterns. Extremely strong (+4) in the gut epithelium, moderate (+2) in parenchyma, and strong (+3) in muscularis and tegumental tubercles (Mo2Ab), suggesting tissue-specific enzymatic activity. Scale bars: 200 and 50 µm.

The immuno-phosphatase technique was used to stain paraffin sections of *S. mansoni* worms (Figure 2). The negative control (Figure 2A) validated the specificity of the phosphatase immunostaining by the absence of non-specific binding or endogenous activity. In Figure 2B, the positive signal in tegumental tubercles and parenchyma suggested robust enzymatic activity potentially involved in nutrient processing or immunomodulation. Figure 2C and D show differential expression levels, reflecting variation in phosphatase activity distribution, possibly linked to metabolic zones or antibody accessibility. The gut showed extremely strong (+4) reactivity, muscularis and tegumental tubercles showed strong (+3) reactivity, while parenchyma showed moderate (+2) reactivity, consistently demonstrating enzymatic labeling and reinforcing their metabolic and immunological relevance in the host-parasite interaction.

The intensity and distribution of schistosomal antigens within worm sections obtained using different MoAb and immune-phosphatase techniques are summarized in Table 1. All MoAb showed extreme phosphatase reaction in the gut epithelium (4+), and strong reaction (3+) in the tegumental tubercles. Different phosphatase reactivity was documented in the other organelles. Mo3Ab showed that no reactions were detected in other worm organelles. Mo2Ab and Mo4Ab showed identical intensity and distribution of schistosomal antigens, tegument, and subsegmental musculature, exhibiting strong reactions (3+). In contrast, the cell bodies and parenchyma had moderate staining (2+), and the gonads showed weak reactions (1+). Mo1Ab showed different phosphatase activity than Mo2Ab and Mo4Ab (Table 1).

**Table 1:** Anatomic localization of schistosomal antigens in sections of *S. mansoni* worms using different monoclonal antibodies (MoAb) using immuno-phosphatase staining.

MoAb*	Tubercles	Tegument	Muscularis	Cell Body	Parenchyma	Gut	Gonads
Mo1Ab	3+	3+	2+	1+	-ve	4+	2+
Mo2Ab	3+	3+	3+	2+	2+	4+	1+
Mo3Ab	3+	-ve	-ve	-ve	-ve	4+	-ve
Mo4Ab	3+	3+	3+	2+	2+	4+	1+

Produced from *S. mansoni* infected mice; -ve: no reaction; 1+: weak reaction; 2+: moderate reaction; 3+: strong reaction; 4+: extreme reaction. Three sections from the *S. mansoni* worm were examined and scored visually for each MoAb.

### Intact worms

**Figure 3:**
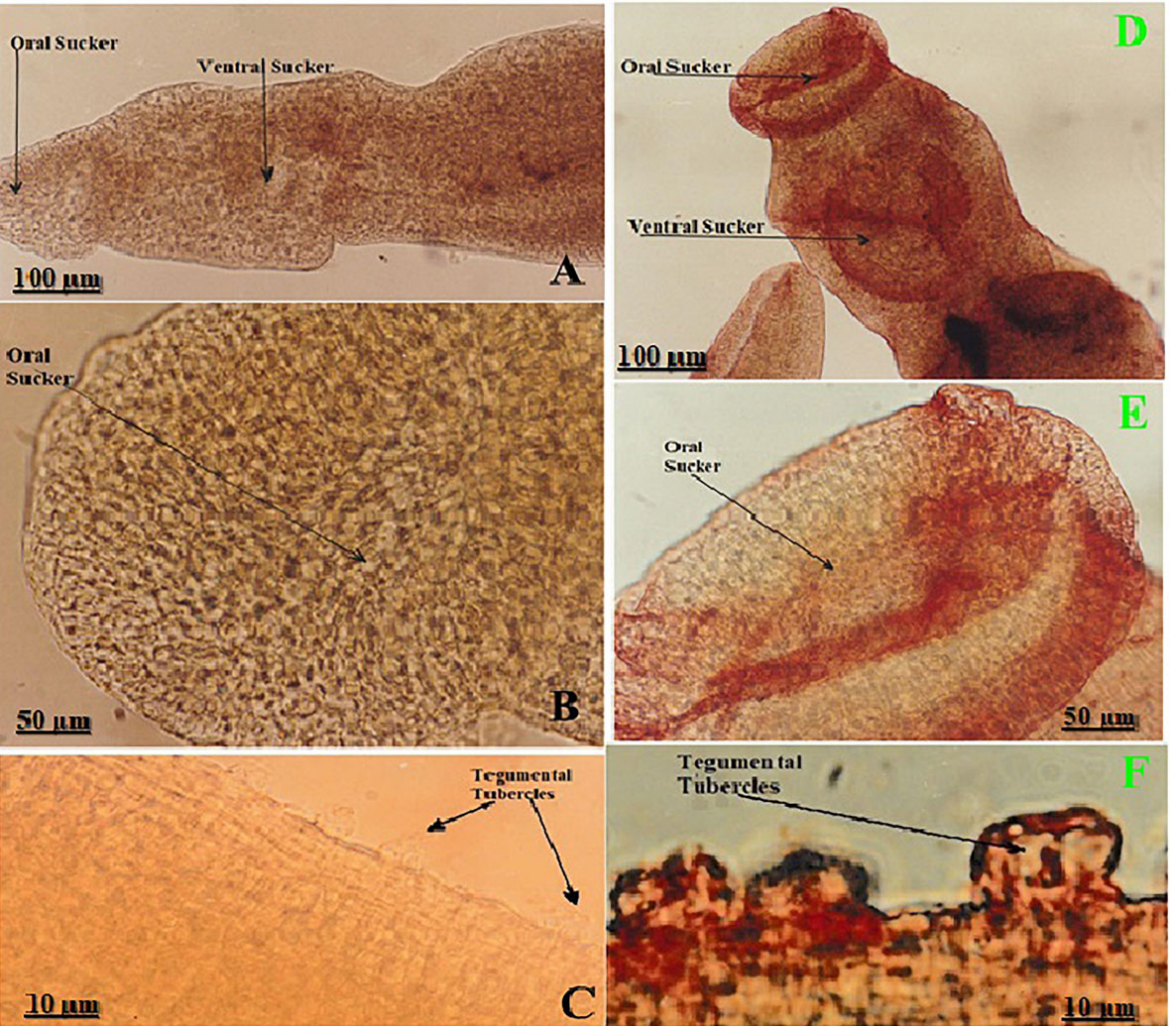
Immunohistochemical detection of peroxidase activity in intact *Schistosoma mansoni* worms. **A**-**C**, Negative control sections showing no detectable peroxidase reaction in the oral and ventral suckers (**A**: 100×, **B**: 400×) and tegumental tubercles (**C**: 1000×). **D**-**F**, Positive peroxidase immunostaining using monoclonal antibody Mo3Ab. The oral and ventral suckers and the tegumental tubercles exhibited strong (+3) staining (**D**: 100×, **E**: 400×, **F**: 1000×). These regions are known for dynamic interaction with host tissues and secretion of immunologically active enzymes. Scale bars: 100, 50, and 10 µm.

The strong endogenous phosphatase on the worm's surface masked the rest of the worm's organelles when formalin-fixed intact *S. mansoni* worms were used. The immuno-peroxidase technique was performed, showing no endogenous peroxidase on the worm surface. Immunohistochemical detection of peroxidase activity in intact *S. mansoni* worms is shown in Figure 3. Negative control images (Figure 3A-C) confirmed antibody specificity and ruled out non-specific or endogenous peroxidase activity. Strong peroxidase activity (+3) is shown in Figure 3D-F, indicating enzymatic expression in critical contact sites (oral sucker, ventral sucker, and tegument), all of which are central to host interaction, attachment, and feeding. High magnification in Figure 3F (1000×) clearly illustrates active enzyme localization within the tegumental tubercles, suggesting functional significance in host interface modulation. Staining of the intact *S. mansoni* worms showed the same peroxidase reactivity in the four different MoAb; they exhibited strong peroxidase reaction (3+) on the dorsum tubercles and suckers, either oral or ventral. The gynecophoric canal had a weak peroxidase reaction (1+).

### Cross-reactivity of different MoAb within schistosomula and intact cercariae of *S. mansoni*

**Figure 4:**
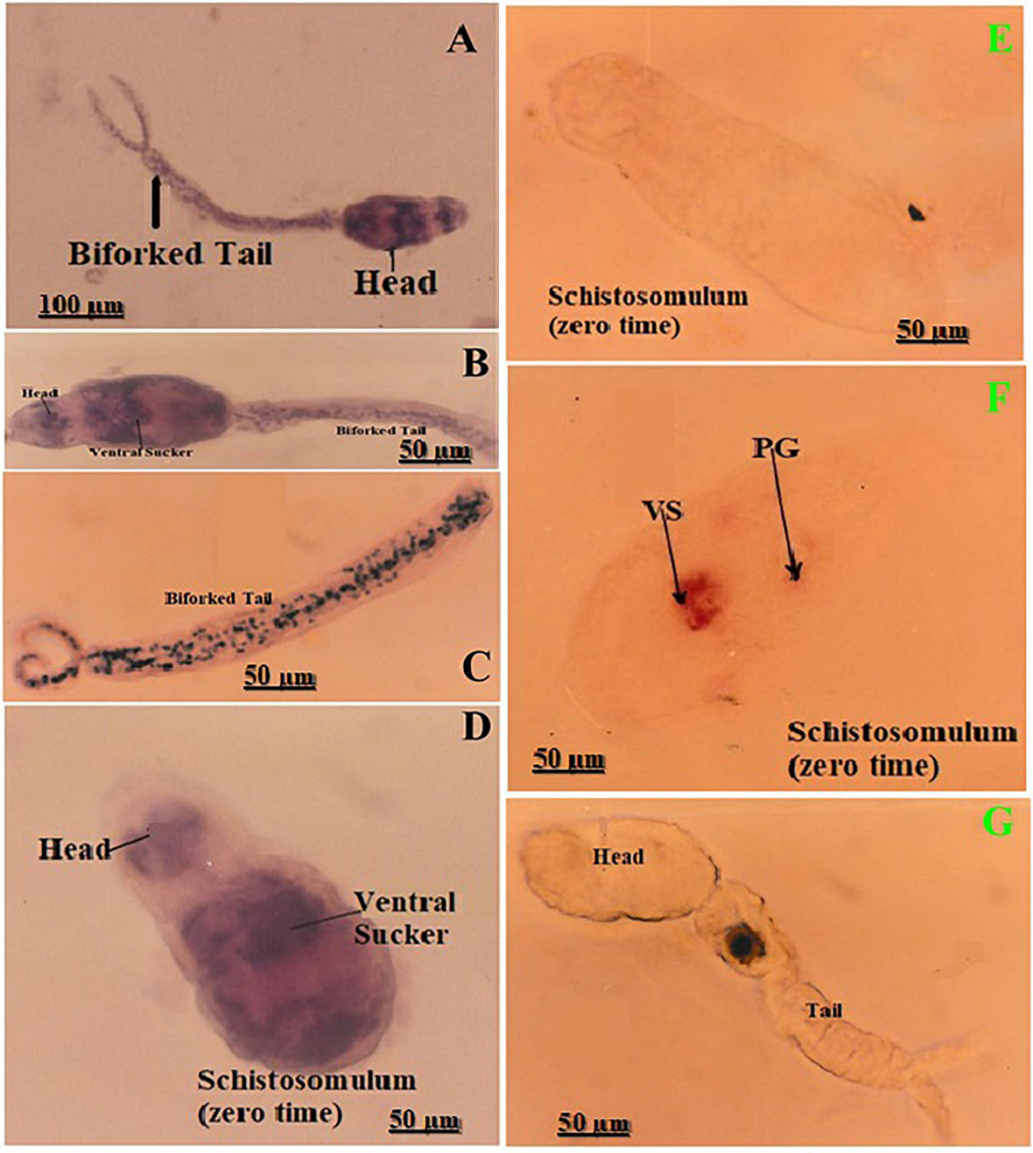
*Schistosoma mansoni* larvae: morphology and localization of a peroxidase-reactive antigen immediately after transformation (time zero). **A**-**C**, Intact cercariae (HE; **A**, 100×; **B**-**C**, 400×) showing the head, ventral sucker, and the characteristic bifurcated tail. **D**, Newly transformed schistosomulum (time zero; HE, 400×) with defined head and ventral sucker. **E**-**G**, localization of a peroxidase-reactive antigen immediately after transformation (time zero). **E**, Negative-control schistosomulum (time zero; 400×) incubated without primary antibody, showing no peroxidase reaction. **F**, Schistosomlum (time zero; 400×) probed with the anti-peroxidase monoclonal antibody Mo1Ab showing a strong positive signal in the penetration glands (PG) and ventral sucker (VS). **G**, Intact cercaria (400×) probed with Mo1Ab showing no detectable peroxidase reaction in either head or tail. Scale bars: 100 and 50 µm.

*S. mansoni* intact schistosomula, cercariae, and the biforked tail of cercaria after transformation were stained with HE to show their structures (Figure 4A-D). The immuno-peroxidase staining was performed on intact cercariae and schistosomula (time zero), using the panel of *S. mansoni* MoAb. No peroxidase reaction on the schistosomulum (time zero) was found when using myeloma cell ascites (negative control), supporting antibody specificity. MoAb had peroxidase reactions within the schistosomulum in the oral sucker and penetration glands; cercariae showed negative peroxidase reactions (Figure 4E-G). The peroxidase signal was absent in intact cercariae (Figure 4G), but appeared immediately after transformation in schistosomula (Figure 4F), indicating a rapid, stage-specific unmasking/secretion or redistribution of the antigen at time zero. The immune reactions for various MoAb with intact schistosomula showed similar peroxidase activity in both the ventral sucker and penetration gland, exhibiting a strong peroxidase reaction (3+). In contrast, cercariae displayed negative peroxidase reactions.

### Cross-reactivity of *S. mansoni* MoAb within different tissues of *S. haematobium* worms

**Figure 5:**
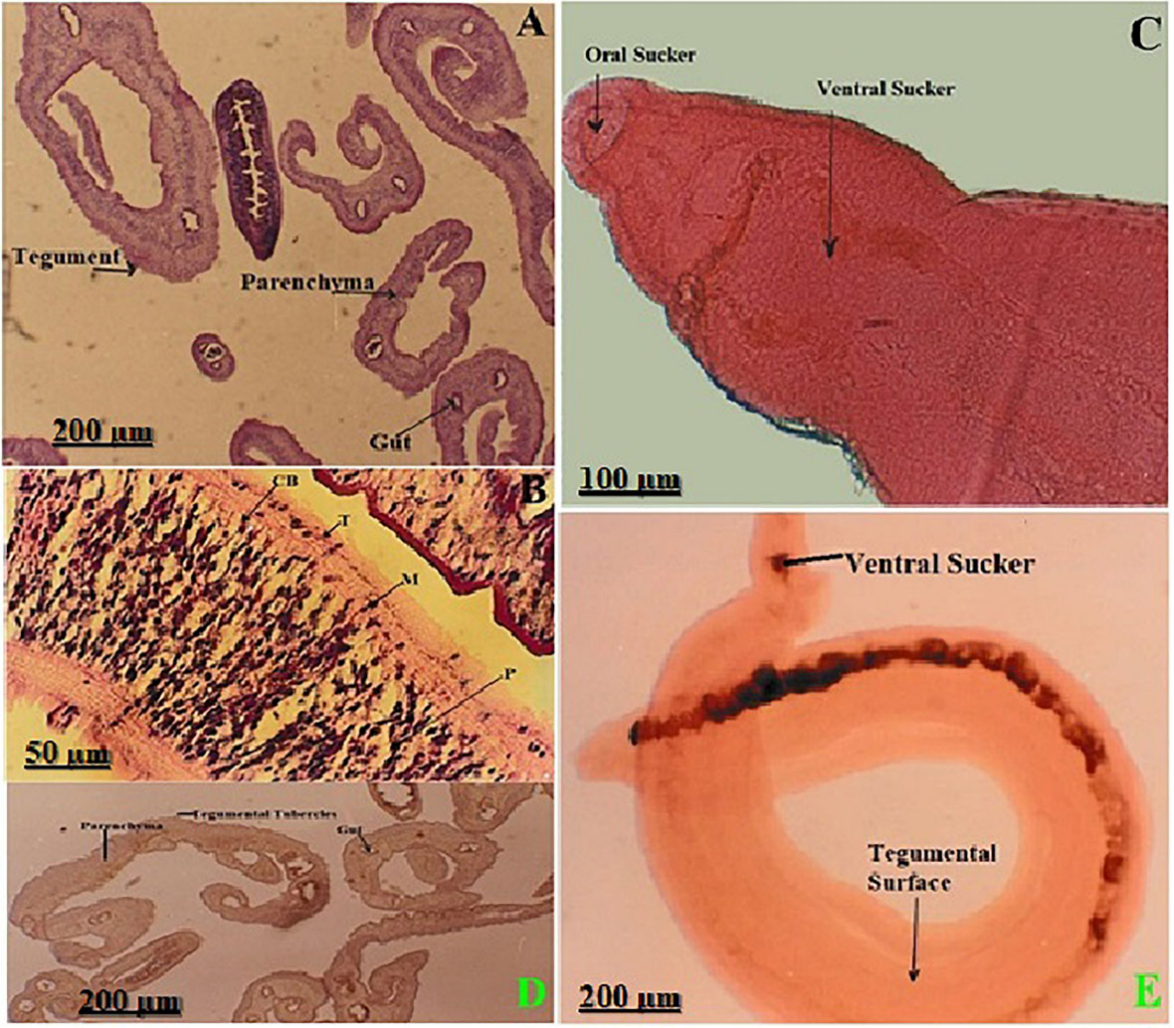
*Schistosoma haematobium* worms: histological architecture and peroxidase reactivity. **A** and **B,** Transverse sections of adult worms (HE; **A**, 40×; **B**, 400×) showing the parenchyma (P), tegument (T), muscularis (M), cell bodies (CB), and gut. **C**, Intact adult worms (HE, 100×) showing oral and ventral suckers. **D**, Section showing weak positive peroxidase reactivity in the gut, tegument, and tegumental tubercles (monoclonal antibody Mo4Ab, 40×). **E**, Entire adult male worm preparation showing weak peroxidase reaction on tegumental surface and ventral sucker (Mo4Ab, 40×). Scale bars: 200, 100, and 50 µm.

HE staining of *S. haematobium* worms (gut, parenchyma, tegument, muscularis, and cell bodies) is shown in Figure 5A and B with well-stained cellular details. Intact *S. haematobium* worms showed the tegumental surface and suckers (Figure 5C).

The immuno-peroxidase technique was used to stain paraffin sections of *S. haematobium* and intact worms. A weak peroxidase reaction (1+) was found in the worm gut (Figure 5D). The intensity of the immuno-peroxidase reaction using different MoAbs is shown in Table 2. Mo1Ab and Mo3Ab had moderate peroxidase reaction (2+) in the gut epithelium, while Mo2Ab and Mo4Ab had weak peroxidase reaction (1+). A weak positive reaction (1+) was exhibited in tegumental tubercles with all MoAbs, and no reaction was documented in the gynecophoric canal. No staining was seen in the tegument. A weak peroxidase reaction was revealed on the intact tegumental surface and suckers (Figure 5E).

**Table 2:** Distribution and intensity of schistosomal antigens in sections of *S. haematobium* worms and intact worms examined with different monoclonal antibodies (MoAb) using immuno-peroxidase staining.

MoAb*	Paraffin sections			Intact worms		
	Tegument	Gut	Teg. Tub.	Teg. Tub.	Suckers	GC
Mo1Ab	-ve	2+	1+	1+	1+	-ve
Mo2Ab	-ve	1+	1+	1+	1+	-ve
Mo3Ab	-ve	2+	1+	-ve	1+	-ve
Mo4Ab	-ve	1+	1+	1+	1+	-ve

Produced from *S. mansoni* infected mice; -ve: no reaction; 1+: weak reaction; 2+: moderate reaction; Teg. Tub.: tegumental tubercles; GC: gynecophoric canal. Three sections from each worm were examined and scored.

### Cross-reactivity of *S. mansoni* MoAb in the intermediate host of *S. mansoni* (*Biomphalaria sp.*) and *S. haematobium* (*Bulinus sp.*)

**Figure 6:**
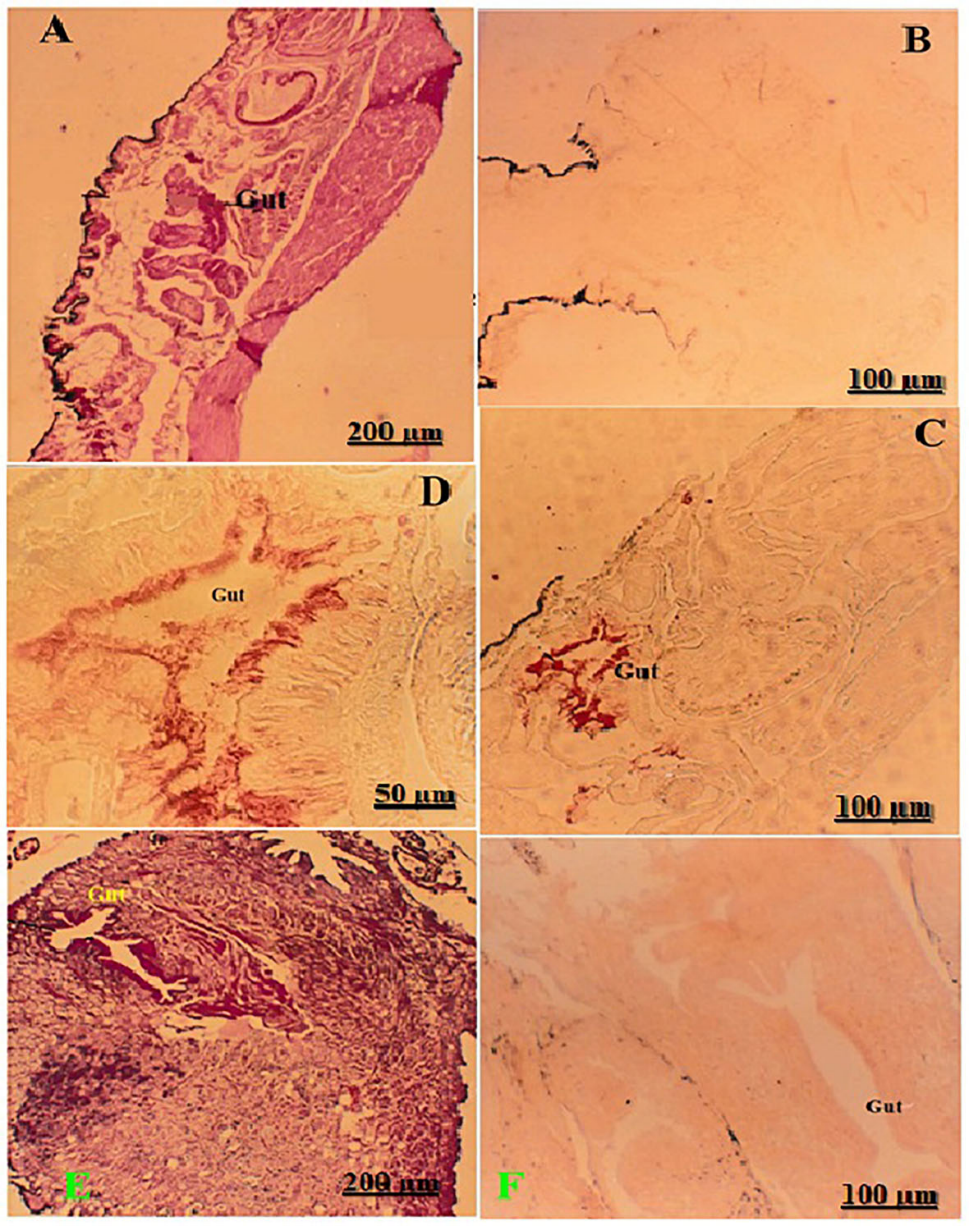
Histological sections of infected snail intermediate hosts (*Biomphalaria sp.* and *Bulinus sp.*) showing gut morphology and peroxidase reactivity. **A**, Sections of *Biomphalaria sp.* snail (shedding cercariae) showing the gut (HE, 40×). **B**, No peroxidase reaction (negative control, 100×). **C** and **D**, positive peroxidase reaction in the gut (monoclonal antibody Mo4Ab; **C**, 100× and **D**, 400×). **E** and **F**, Sections of *Bulinus sp.* snail (shedding cercariae) showing the gut (**E**, HE, 40×) and negative peroxidase reaction in the gut (**F**, Mo4Ab, 100×). Scale bars: 200, 100, and 50 µm.

The HE stain was applied to sections of infected *Biomphalaria* and *Bulinus* sp. snails that excreted cercariae to show the snail's structure (Figure 6A and E). The immuno-peroxidase technique was performed on normal and infected *Biomphalaria* sp. snails. There was no peroxidase reaction in the normal tissues of the snail using MoAb. The infected snails had no peroxidase reaction with myeloma cell ascites. MoAb exhibited extremely strong peroxidase reaction (4+) within the gut of the infected snails Figure 6C and D. There was a negative peroxidase reaction in the gut of infected *Bulinus* sp. snails, as indicated by the lack of MoAb reactivity (Figure 6F). The strong staining in the gut of *Biomphalaria* (Figure 6C and D) *versus* the absence of reaction in *Bulinus* (Figure 6F) may suggest differential expression or availability of peroxidase-like antigens between snail hosts, representing species-specific peroxidase activity. These findings may relate to host-parasite compatibility, as *Biomphalaria* snails are the primary hosts for *S. mansoni*, whereas *Bulinus* is the host for *S. haematobium*, indicating potentially distinct handling of oxidative stress in their digestive epithelia during infection. The inclusion of negative control staining (Figure 6B) provided confidence in the specificity of Mo4Ab.

## Discussion

Despite the extensive worldwide efforts to control schistosomiasis, the disease continues to spread to new areas. The main challenge lies in the diagnosis, highlighting the need to develop practical and scalable techniques. Serological examinations, used to screen for anti-schistosomal antibodies, have limited efficacy due to the widespread presence of antibodies in the population from past infections. Thus, there is a pressing need to identify the most effective antigen for use in ELISA to accurately diagnose patients from endemic areas and monitor their treatment progress (8,9).

In this study, the four *S. mansoni* MoAb were prepared using the hybridoma technique, and selected MoAb were chosen according to the high reactivity to WASP antigen preparation using the ELISA technique. The specificity of these monoclonal antibodies was evaluated against various life cycle stages of *S. mansoni* and *S. haematobium* worms, as well as their intermediate hosts. Histological and immunohistochemical analyses were performed to characterize the structural and enzymatic profiles of *S. mansoni* adult worms using H&E, phosphatase, and peroxidase staining methods. The findings provided insight into the parasite's internal anatomy, tegumental features, and functional enzymatic activity at key host-parasite interfaces. A specific localization and reactivity of the four selected MoAb was also provided.

The HE-stained sections displayed the distinct internal architecture of *S. mansoni*. Transverse and longitudinal views displayed prominent tegumental tubercles, a well-organized muscularis layer, the parenchymal tissue, and a centrally located gut with a visible lumen. The ventral sucker and gynecophoric canal were observed in longitudinal sections, indicating sexual dimorphism and the specific role of the male in harboring the female worm. These morphological features are consistent with previous descriptions of adult schistosomes and support a highly adapted structure for vascular attachment and nutrient absorption. The tegument, in particular, has been reported to play a dynamic role in host immune evasion, nutrient uptake, and sensory function. The parenchyma appears densely populated with cellular and glandular structures, while the gut is lined with absorptive cells essential for processing host-derived macromolecules (18,19).

The current study used immunohistochemical staining to assess the anti-phosphatase activities of various monoclonal antibodies. This revealed positive phosphatase activity in the gut, tegumental tubercles, parenchyma, and muscularis across different sections. In contrast, the negative control showed no staining, confirming the specificity of different MoAb. These observations indicate that phosphatases are highly expressed in metabolically active tissues, particularly those involved in nutrient absorption (gut) and host interface interactions (tegument). Tegumental phosphatases may participate in phosphate turnover, immune modulation, and enzymatic detoxification mechanisms that are vital for parasite survival in the hostile intravascular environment (20,21). The muscularis layer's enzymatic activity may be related to energy regulation required for motility and sucker function.

Targeted peroxidase immunostaining demonstrated strong enzymatic localization at the oral sucker, ventral sucker, and tegumental tubercles, whereas negative controls displayed no signal. These suckers are key attachment and feeding structures, frequently exposed to host-derived oxidative stress. The tegumental peroxidase expression suggests a protective antioxidant role, enabling schistosomes to withstand reactive oxygen species produced by host immune responses. This finding aligns with reports that peroxidase enzymes are integral to tegumental antioxidant defenses, facilitating membrane maintenance, immune evasion, and redox regulation at the host-parasite interface (18,21). The enzyme's presence in oral structures may also support tissue penetration and localized detoxification during feeding.

The transformation of *S. mansoni* cercariae into schistosomula is a rapid process characterized by shedding of the cercarial glycocalyx, discharge of acetabular/penetration gland contents, and extensive remodeling of the anterior end without growth (22). Our immunohistochemical data place a peroxidase-reactive antigen squarely within the penetration glands and ventral sucker at time zero, while intact cercariae were non-reactive. This spatial and temporal pattern suggests that i) the enzyme (or enzyme-containing complex) is either stored in the penetration glands and released/activated upon transformation or ii) epitopes become immunologically accessible only after gland discharge and tegumental reorganization. Developmental transcriptomic and single-cell atlases reveal significant stage-specific regulation of redox genes during transition from cercaria-to-schistosomulum. This regulation is particularly evident in and around the penetration glands as well as in the region anterior to the ventral sucker (22,23). Our finding that MoAb marks these very structures at the first moments after transformation fits these multi-omic observations and supports a model in which antioxidant/peroxidase activity is deployed at the host-parasite interface from the outset of invasion.

The present data revealed that peroxidase-reactive antigens are strongly exposed at the moment of cercaria-to-schistosomulum transformation in *S. mansoni*, only weakly detectable on the tegument and gut of adult *S. haematobium*, and differentially detectable in the guts of the compatible (*Biomphalaria*) *versus* incompatible (*Bulinus*) snail hosts. These patterns align with modern multi-omic atlases and redox-biology studies, highlighting how schistosomes reprogram antioxidant defenses in a stage- and tissue-specific manner to endure the intense oxidative stress associated with invasion and early intra-mammalian development (22,24,25). The strong reaction in schistosomula penetration glands and the ventral sucker is consistent with the rapid secretion/unmasking of invasion-associated proteins immediately after skin penetration, as mapped by recent cellular atlases of the cercariae-schistosomulum transition and early intra-mammalian development (22,26). In adults, the staining of *Schistosoma haematobium* with peroxidase is weak and primarily localized to the surface of the tegument, the tegumental tubercles, and the gut. This observation aligns with proteomic and transcriptomic evidence, indicating that while the tegument maintains a strong antioxidant defense, the highest oxidative stress occurs earlier during the processes of transformation and migration (22,24,26). Finally, our snail data revealed clear, gut-localized peroxidase reactivity in *Biomphalaria* (*S. mansoni*), but not in *Bulinus* (*S. haematobium*), suggesting species-specific antioxidant responses and/or parasite-driven modulation of snail redox balance. Recent studies on snail-schistosome interactions reveal significant immune-metabolic and physiological changes in infected snails. These include alterations in oxidative pathways, with compatibility playing a crucial role in shaping the nature and extent of these responses (25,27).

## Conclusion

Together, these histological and enzymatic analyses offer a comprehensive view of the functional morphology of *S. mansoni*. The tegument, gut, and suckers not only serve structural and physiological roles but also exhibit targeted phosphatase and peroxidase activity, underlining their metabolic and immunological importance. These findings highlight potential tissue- and enzyme-specific targets for diagnostic, vaccine, or drug development strategies against schistosomiasis. In summary, MoAbs directed to *S. mansoni* SWAP are valid and valuable in the immune-diagnosis of schistosomiasis, particularly for enhancing sensitivity, specificity, and stage detection. Their integration into diagnostic strategies, especially in endemic, low-resource regions, can significantly improve disease surveillance and control efforts.

## Data Availability

All data generated or analyzed during this study are included in this published article.
